# Health Care Burden of Bronchopulmonary Dysplasia Among Extremely Preterm Infants

**DOI:** 10.3389/fped.2019.00510

**Published:** 2019-12-12

**Authors:** Meredith E. Mowitz, Rajeev Ayyagari, Wei Gao, Jing Zhao, Alexandra Mangili, Sujata P. Sarda

**Affiliations:** ^1^Division of Neonatology, College of Medicine, University of Florida, Gainesville, FL, United States; ^2^Analysis Group Inc., Boston, MA, United States; ^3^Shire, A Takeda Company, Lexington, MA, United States

**Keywords:** bronchopulmonary dysplasia, comorbidities of prematurity, cost analysis, health care resource utilization, neonatology

## Abstract

**Background:** Infants born extremely preterm are at high risk of developing bronchopulmonary dysplasia (BPD). This study aimed to assess the incremental health care burden of BPD and associated comorbidities among extremely preterm infants in the United States.

**Methods:** Health service claims in the Premier Perspective database were retrospectively analyzed for infants born at ≤28 weeks gestation who were admitted to neonatal intensive care during birth hospitalization and survived to a postmenstrual age of ≥36 weeks. Gestational age (GA) at birth and BPD status of infants was determined based on International Classification of Diseases, Ninth Revision, Clinical Modification (ICD-9-CM) codes recorded in the database.

**Results:** Of the 12,017 infants included, 4,904 (40.8%) had BPD. BPD increased with decreasing GA: 67.4% of infants born at <24 weeks GA had BPD vs. 28.7% of those born at 27–28 weeks. Infants with BPD had significantly longer hospital stays following birth than those without (mean [standard deviation (SD)] 102 [34] vs. 83 [24] days, respectively, *P* < 0.001), and incurred higher total charges (mean [SD] $799,499 [$535,528] vs. $588,949 [$377,137], respectively, *P* < 0.001). Mean total charges incurred during index hospitalization decreased as GA at birth increased, with GA having a bigger effect than presence or absence of BPD. During their first year, infants with BPD had a higher in-hospital late mortality rate than those without (1.9 vs. 0.6%), and were more likely to have two or more hospital encounters following birth hospitalization (58.0 vs. 48.2%). Among infants who had two or more encounters after discharge, those with BPD experienced a higher percentage of pulmonary symptoms than those without (46.3 vs. 38.9%). Comparison with infants who did not have BPD, retinopathy of prematurity, or intraventricular hemorrhage showed that BPD is the main complication contributing to increased length of stay, costs, in-hospital mortality, and additional health care encounters.

**Conclusion:** BPD is a key contributor to the large health care burden associated with extremely preterm birth. However, GA at birth has a bigger effect on health care costs for extremely preterm infants than the presence of BPD.

## Introduction

Each year, >28,000 babies in the United States are born extremely preterm (<28 weeks gestation) (1). Survival of extremely preterm infants has steadily increased in recent years owing to advances in maternal and neonatal care (2, 3). In academic centers in the United States, the majority of infants born at a gestational age (GA) of ≥24 weeks will survive until discharge from their birth hospital (2). However, infants who survive extremely preterm birth are at increased risk for multiple morbidities that are associated with significant clinical burden during the newborn period, and require neonatal intensive care and long hospital stays (4, 5).

One of the most prevalent and clinically significant morbidities among infants born preterm is bronchopulmonary dysplasia (BPD), which can develop from the disruption of lung development (6–8). An increase in the incidence of BPD in extremely preterm infants in the United States has been reported, and this may be due in part to the increased survival of infants born at younger GAs (2). Infants with BPD require significantly longer hospital stays at birth and have a higher risk of delayed discharge than those without BPD (9). Because they may experience pulmonary issues for a long time following the neonatal period, infants with BPD are also more likely to require rehospitalization and make increased use of health care services during their first years of life (10). Over the long term, preterm infants who develop BPD are more commonly affected by chronic respiratory problems, cardiovascular impairment, and neurodevelopmental delay than those who do not develop BPD (8).

Other morbidities of extremely preterm birth, including intraventricular hemorrhage (IVH) and retinopathy of prematurity (ROP), may also have adverse outcomes. IVH is a notable cause of brain injury and is associated with developmental delay and neurosensory impairment; ROP is a significant cause of visual impairment, requiring specialized treatment in severe cases to mitigate permanent vision loss (11, 12). Because the incidence and severity of these morbidities increase with decreasing GA at birth, the same infants who develop BPD are also at high risk of developing ROP and IVH, adding to the already high burden of health care costs and resource utilization associated with extremely preterm infants (13, 14).

Insights into the health care burden of infants with BPD can help with management of medical care resources, counseling of parents, and development of effective treatments to improve patient outcomes and reduce care costs. The aim of this study was to assess the incremental health care burden of BPD and its interaction/association with comorbidities such as ROP and IVH among extremely preterm infants in urban centers in the United States, using data from the Premier Perspective health care services database. Presence or absence of BPD and comorbid conditions was determined based on diagnostic codes recorded in the database.

## Materials and Methods

### Data Source

This study was a retrospective analysis of the Premier Perspective database, which contains patient data in de-identified records from inpatient and outpatient visits to ~500 geographically diverse hospitals in the United States. Health care service utilization records in the database contain information on patients' demographic and clinical characteristics and details of billed services received during hospital visits, such as medications and therapeutic and diagnostic procedures (15).

### Patient Selection

**Figure 1:**
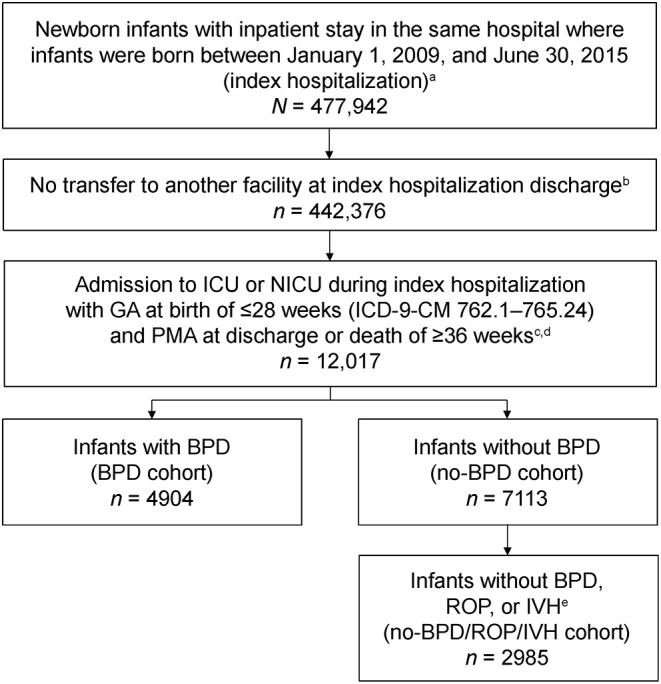
Selection of the analysis cohort from the Premier Perspective claims database. BPD, bronchopulmonary dysplasia; GA, gestational age; ICD-9-CM, International Classification of Diseases, Ninth Revision, Clinical Modification; ICU, intensive care unit; IVH, intraventricular hemorrhage; NICU, neonatal intensive care unit; PMA, postmenstrual age; ROP, retinopathy of prematurity. ^a^Index hospitalization was defined as the newborn infant's first hospitalization. ^b^Only infants discharged to home or those who died during index hospitalization were included in the analysis. ^c^Only patients with non-zero costs during index hospitalization were included. ^d^PMA at discharge was calculated as the GA at birth plus the length of stay during index hospitalization until discharge. ^e^Presence of morbidities was identified from ICD-9-CM codes recorded during index hospitalization.

Data were retrieved on infants born between January 1, 2009 and June 30, 2015, who had an inpatient stay during their birth hospitalization of ≥1 day (index hospitalization). Of these infants, those selected for analysis were born at a GA of ≤28 weeks, admitted to the neonatal intensive care unit (NICU) during their index hospitalization, and discharged to home or died in the hospital after the postmenstrual age (PMA) of ≥36 weeks (Figure 1). To ensure that all birth hospitalization charges were captured, infants were only included if they had been discharged to home or died in the same hospital during index hospitalization, and did not transfer to another hospital. Infants who had zero costs during index hospitalization were excluded. GA at birth was determined from the highest classification code for GA [International Classification of Diseases, Ninth Revision, Clinical Modification (ICD-9-CM)] recorded during index hospitalization (Supplementary Table 1). Although infants born at 28 completed weeks of gestation are outside the definition of extremely preterm, they were included in the analysis because they were captured under the ICD-9-CM code for 27–28 weeks GA. PMA at discharge or death was calculated as the GA at birth plus the number of weeks of index hospitalization until discharge or death.

### Analysis

Presence of BPD, ROP, IVH, and other select comorbidities were determined from ICD-9-CM codes recorded in the database during index hospitalization (Supplementary Table 2). Infants were categorized into two groups for analysis based on the presence or absence of BPD. As BPD and chronic lung disease (CLD) are coded using the same ICD-9-CM codes, the BPD cohort included both. For infants without BPD (no-BPD cohort), a subgroup was created to analyze those who also did not have ROP or IVH (no-BPD/ROP/IVH cohort; Figure 1). Statistical comparisons between cohorts were conducted for the study outcomes with the Wilcoxon rank-sum test.

### Outcomes

Length of stay (LOS), charges, and costs during index hospitalization were determined for each cohort, as were in-hospital mortality and rates of readmission during the first year after index hospitalization. Charges were calculated from coding of billable items for each patient, on the basis of health care providers' listed charges. Costs were computed from the actual cost to treat each patient, including supplies, labor, and depreciation of equipment; total costs were calculated as the sum of variable costs (direct) and fixed costs (overhead). Charges and costs were inflated to 2015 US dollars using the US Medical Service Consumer Price Index. Pulmonary symptoms reported during the first year of life were analyzed for infants who had at least two additional health care encounters of any type (outpatient visit, emergency department visit, or hospital readmission associated with a pulmonary diagnosis) following index hospitalization. Pulmonary-related medication use associated with outpatient visits during the first year was analyzed for infants who had at least two additional health care encounters following index hospitalization. Pulmonary symptoms and medications were identified from Current Procedural Terminology and ICD-9-CM codes, and by searching for the generic medication name in standard charge code descriptions (Supplementary Tables 2,3).

## Results

### Patient Characteristics

A total of 12,017 infants born at ≤28 weeks GA were included in the analysis. Of these, 4,904 (40.8%) had BPD and 7,113 (59.2%) did not have BPD (Table 1). Of the infants who did not have BPD, 2,985 (42.0%) also did not have ROP or IVH. BPD increased with decreasing GA: 353 of 524 (67.4%) infants born at <24 weeks GA had BPD vs. 1,747 of 6,091 (28.7%) infants born at 27–28 weeks GA.

**Table 1:** Demographics and clinical characteristics of extremely preterm infants with and without BPD at the time of index hospitalization (*N* = 12,017).

	**BPD**	**No BPD**	**No BPD, ROP, or IVH**
**Characteristic, *n* (%)**	***n* = 4,904**	***n* = 7,113**	***n* = 2,985**
Female	2,328 (47.5)	3,523 (49.5)	1,483 (49.7)
Race			
White	2,124 (43.3)	3,183 (44.7)	1,313 (44.0)
Black	1,333 (27.2)	1,787 (25.1)	762 (25.5)
Other	1,424 (29.0)	2,119 (29.8)	899 (30.1)
GA at birth^a^			
<24 weeks	353 (7.2)	171 (2.4)	23 (0.8)
24 weeks	768 (15.7)	517 (7.3)	96 (3.2)
25–26 weeks	2,036 (41.5)	2,081 (29.3)	657 (22.0)
27–28 weeks	1,747 (35.6)	4,344 (61.1)	2,209 (74.0)
PMA at discharge^b^			
36–38 weeks	1,828 (37.3)	4,509 (63.4)	2,130 (71.4)
39–41 weeks	1,765 (36.0)	1,859 (26.1)	649 (21.7)
42–44 weeks	760 (15.5)	493 (6.9)	137 (4.6)
45–47 weeks	292 (6.0)	145 (2.0)	41 (1.4)
≥48 weeks	259 (5.3)	107 (1.5)	28 (0.9)
Select comorbidities^c^			
ROP^d^	2,751 (56.1)	3,215 (45.2)	0 (0.0)
Stage 1	937 (19.1)	1,375 (19.3)	0 (0.0)
Stage 2	988 (20.1)	958 (13.5)	0 (0.0)
Stage 3	571 (11.6)	388 (5.5)	0 (0.0)
Stage 4	4 (0.1)	6 (0.1)	0 (0.0)
Stage 5	2 (0.0)	0 (0.0)	0 (0.0)
Unspecified	249 (5.1)	488 (6.9)	0 (0.0)
IVH^d^	1,652 (33.7)	1,841 (25.9)	0 (0.0)
Grade 1	680 (13.9)	807 (11.3)	0 (0.0)
Grade 2	398 (8.1)	453 (6.4)	0 (0.0)
Grade 3	222 (4.5)	226 (3.2)	0 (0.0)
Grade 4	260 (5.3)	224 (3.1)	0 (0.0)
Unspecified	92 (1.9)	131 (1.8)	0 (0.0)
Necrotizing enterocolitis	511 (10.4)	519 (7.3)	155 (5.2)
Bacterial sepsis (early or late onset)	2,589 (52.8)	2,677 (37.6)	909 (30.5)
Patent ductus arteriosus	2,997 (61.1)	3,529 (49.6)	1,294 (43.4)

*BPD, bronchopulmonary dysplasia; GA, gestational age; ICD-9-CM, International Classification of Diseases, Ninth Edition, Clinical Modification; IVH, intraventricular hemorrhage; PMA, postmenstrual age; ROP, retinopathy of prematurity*.

a *GA was determined on the basis of the greatest GA identified in the index hospitalization*.

b *PMA at discharge was calculated as the GA plus the number of weeks during the hospitalization until discharge*.

c *Comorbidities were identified from ICD-9-CM codes*.

d *ROP stage and IVH grade are based on the most severe stage/grade identified during index hospitalization*.

The incidence and severity of ROP and IVH were, respectively, higher in infants with BPD than in those without (Table 1). Among infants with BPD, 56.1% had ROP and 33.7% had IVH, whereas among those without BPD, 45.2% had ROP and 25.9% had IVH. For infants who were born at <24 weeks GA and survived to 36 weeks PMA, a similar pattern of worse outcomes was observed among infants with BPD vs. those without (data not shown in Table 1). Among 353 infants who were born at <24 weeks GA, survived to 36 weeks PMA, and had BPD, 75.4% had ROP, 58.4% had IVH, and 3.7% had late death in the hospital within a year of birth. The corresponding percentages among infants of <24 weeks GA who did not have BPD (*n* = 171) were ROP 71.3%, IVH 56.7%, and late mortality 2.9%. Where ROP stage was specified, the incidence of severe ROP (stages 3–5) among infants of all included GAs was 23.1% in the BPD cohort and 14.4% in the no-BPD cohort (*P* < 0.001). The incidence of severe IVH (grades 3–4) was 30.9% (482/1,560 cases with IVH grade specified) for infants with BPD, and 26.3% (450/1,710) for those without BPD (*P* = 0.004). Other complications that were more common in infants with BPD included necrotizing enterocolitis, bacterial sepsis, and patent ductus arteriosus (Table 1).

### Resource Utilization

Preterm infants who had BPD remained in their hospital of birth significantly longer than those who did not have BPD (mean [standard deviation (SD)] LOS: 102 [34] vs. 83 [24] days, respectively; *P* < 0.001) and those who did not have BPD, ROP, or IVH (mean [SD] LOS: 102 [34] vs. 77 [20] days; *P* < 0.001; Table 2). The LOS for the no-BPD cohort was slightly longer than that for the no-BPD/ROP/IVH cohort (mean [SD] LOS: 83 [24] vs. 77 [20] days, respectively; *P* < 0.001). LOS decreased as GA at birth increased (Table 2).

**Table 2:** Length of stay during index hospitalization and hospital readmission and mortality during 1 year after index hospitalization in extremely preterm infants with or without BPD (*N* = 12,017).

	**BPD**	**No BPD**	**No BPD, ROP, or IVH**
	***n* = 4,904**	***n* = 7,113**	***n* = 2,985**
Mean (SD) length of stay, days			
Full hospitalization^a^	102 (34)^b^	83 (24)^b^	77 (20)^b^
GA <24 weeks	135 (39)	127 (34)	122 (26)
GA 24 weeks	122 (31)	112 (24)	109 (19)
25 ≤ GA ≤ 26 weeks	104 (29)	92 (21)	89 (19)
27 ≤ GA ≤ 28 weeks	85 (29)	73 (18)	72 (17)
ICU/NICU^c^	87 (38)^b^	65 (31)^b^	61 (28)^b^
GA <24 weeks	118 (44)	103 (41)	107 (34)
GA 24 weeks	107 (36)	94 (32)	94 (31)
25 ≤ GA ≤ 26 weeks	89 (35)	74 (29)	72 (28)
27 ≤ GA ≤ 28 weeks	70 (34)	57 (26)	56 (25)
Readmissions and in-hospital late mortality during 1 year after index hospitalization, *n* (%)			
All-cause readmissions	767 (15.6)^b^	737 (10.4)^b^	275 (9.2)^b^
Lung-related readmissions	656 (13.4)^b^	556 (7.8)^b^	214 (7.2)^b^
Lung-related emergency department visits	623 (12.7)^b^	767 (10.8)^b^	296 (9.9)^b^
In-hospital late mortality^d^	93 (1.9)	44 (0.6)	12 (0.4)

*BPD, bronchopulmonary dysplasia; GA, gestational age; ICU, intensive care unit; IVH, intraventricular hemorrhage; NICU, neonatal intensive care unit; ROP, retinopathy of prematurity*.

a *Duration of full hospitalization stay includes length of stay in the NICU*.

b *Statistical comparisons between the BPD and the no-BPD cohorts and between the BPD and the no-BPD/ROP/IVH cohorts for these numbers were conducted using Chi-square tests; resulting P-values were <0.001, except for the comparison between BPD and no-BPD cohorts for lung-related emergency department visits, for which the value was 0.001*.

c *Stay in the ICU/NICU calculated from number of service days with ICU/NICU standard charge codes*.

d *In-hospital mortalities that occurred during encounters following index hospitalization*.

**Figure 2:**
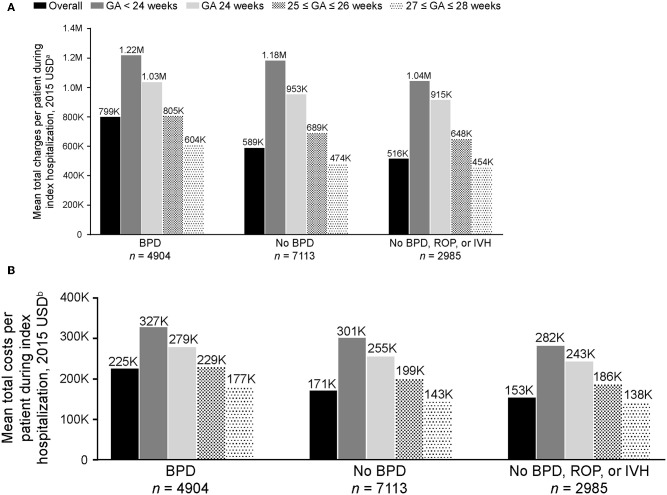
Mean **(A)** total charges and **(B)** costs per patient to treat extremely preterm infants with and without BPD during index hospitalization (*N* = 12,017). Charges and costs inflated to 2015 USD using the US Medical Service Consumer Price Index. BPD, bronchopulmonary dysplasia; GA, gestational age; IVH, intraventricular hemorrhage; ROP, retinopathy of prematurity; USD, United States dollars. ^a^Charge of billable items for each patient. ^b^Actual cost to treat each patient, including supplies, labor, and depreciation of equipment; total costs were calculated as the sum of variable costs (direct) and fixed costs (overhead). The *P*-values for the comparisons of infants with overall BPD vs. no-BPD, and BPD vs. no-BPD/ROP/IVH were <0.001.

On average, preterm infants with BPD incurred higher total costs (mean [SD] $225,204 [$122,365]) than those without BPD (mean [SD] $171,097 [$93,498]; Figure 2). Infants without BPD, ROP, or IVH incurred a mean (SD) total cost of $153,306 ($83,324). Similar trends were seen in charges. Infants with BPD incurred higher total charges (mean [SD] $799,499 [$535,528]) than those without BPD (mean [SD] $588,949 [$377,137]; Figure 2). Infants without BPD, ROP, or IVH incurred the lowest mean (SD) total charge of $515,944 ($327,615), suggesting that BPD may be a bigger driver of costs than ROP or IVH.

Mean total charges and costs incurred during index hospitalization decreased as GA at birth increased (Figure 2). The in-hospital rate of deaths during the first year after index hospitalization was higher for preterm infants with BPD (93/4,904 [1.9%]), compared with those without BPD (44/7113 [0.6%]) and those without BPD, ROP, or IVH (12/2985 [0.4%]; Table 2). Rates of all-cause and pulmonary-related readmissions to the hospital were significantly higher in infants with BPD compared with those without. Emergency room visits for pulmonary-related issues were also higher in infants with BPD (Table 2).

### Pulmonary Symptoms and Pulmonary-Related Medication Use

**Figure 3:**
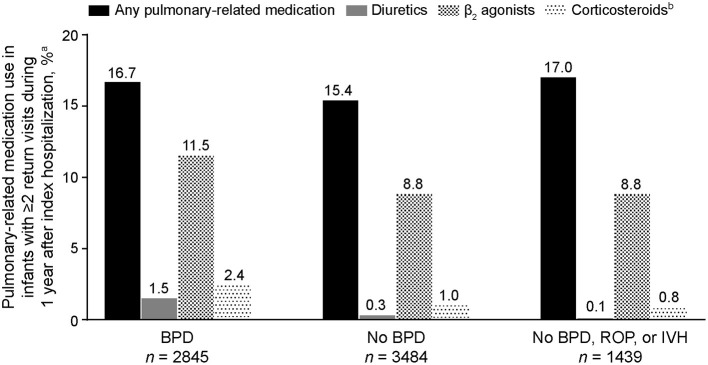
Pulmonary-related medication use during the first year of life in extremely preterm infants who had at least two additional health care encounters following index hospitalization (*n* = 6,329). BPD, bronchopulmonary dysplasia; IVH, intraventricular hemorrhage; ROP, retinopathy of prematurity. ^a^Infants who had at least two encounters (outpatient visit, emergency room visit, or hospital readmission) after their index hospitalization; only pulmonary-related medications reported during outpatient visits are included. ^b^Inhaled formulations.

Of the 12,017 preterm infants included in the analysis, 6,329 returned at least twice for an outpatient visit, emergency department visit, or readmission during the first year following their index hospitalization. It was more common for infants with BPD to have at least two of these additional health care encounters (2,845/4,904 [58.0%]) than for those without BPD (3,484/7,113 [49.0%]; BPD vs. no-BPD *P* < 0.001) and those without BPD, ROP, or IVH (1,439/2,985 [48.2%]; BPD vs. no-BPD/ROP/IVH *P* < 0.001). Among infants who had at least two additional encounters, those with BPD experienced a higher percentage of pulmonary symptoms (1,317/2,845 [46.3%]) than those without BPD (1,356/3,484 [38.9%]; BPD vs. no-BPD *P* < 0.001) and those without BPD, ROP, or IVH (529/1,439 [36.8%]; BPD vs. no-BPD/ROP/IVH *P* < 0.001). Although the five most common pulmonary symptoms/disorders reported were the same in all three cohorts (apnea, dyspnea, bronchiolitis, cough, and upper respiratory tract infections), pneumonia, asthma, reactive airway disease, and hypoxemia were notably more common in infants with BPD (Table 3). Among infants with at least two encounters, pulmonary medication use associated with outpatient visits during the first year after index hospitalization was reported in 474 (16.7%) infants with BPD, 536 (15.4%) infants without BPD, and 244 (17.0%) infants without BPD, ROP, or IVH (Figure 3). β_2_ agonists were the most common pulmonary medications used.

**Table 3:** Incidence of select pulmonary symptoms during 1 year after index hospitalization in preterm infants who had at least two additional health care encounters following index hospitalization (*N* = 6,329)^a^.

	**BPD**	**No BPD**	**No BPD, ROP, or IVH**
**Symptom, *n* (%)**	***n* = 2,845**	***n* = 3,484**	***n* = 1,439**
Any pulmonary symptom	1,317 (46.3)	1,356 (38.9)	529 (36.8)
Dyspnea	578 (20.3)	541 (15.5)	210 (14.6)
Apnea	540 (19.0)	467 (13.4)	173 (12.0)
Bronchiolitis	419 (14.7)	405 (11.6)	157 (10.9)
Cough	403 (14.2)	483 (13.9)	197 (13.7)
Upper respiratory tract infection	402 (14.1)	513 (14.7)	187 (13.0)
Respiratory distress syndrome	352 (12.4)	287 (8.2)	107 (7.4)
Pneumonia	314 (11.0)	279 (8.0)	98 (6.8)
Hypoxemia	276 (9.7)	150 (4.3)	57 (4.0)
Asthma	244 (8.6)	204 (5.9)	90 (6.3)
Reactive airway disease	233 (8.2)	201 (5.8)	89 (6.2)
Wheeze	131 (4.6)	146 (4.2)	61 (4.2)
Tracheostomy complications	121 (4.3)	72 (2.1)	28 (1.9)
Respiratory failure	85 (3.0)	63 (1.8)	23 (1.6)
Tachypnea	59 (2.1)	34 (1.0)	14 (1.0)
Stridor	57 (2.0)	54 (1.5)	18 (1.3)
Bronchitis	41 (1.4)	70 (2.0)	28 (1.9)
Cyanosis	36 (1.3)	35 (1.0)	19 (1.3)
Respiratory syncytial virus	18 (0.6)	21 (0.6)	7 (0.5)

*BPD, bronchopulmonary dysplasia; ICD-9-CM, International Classification of Diseases, Ninth Edition, Clinical Modification; IVH, intraventricular hemorrhage; ROP, retinopathy of prematurity*.

a *Pulmonary symptoms were identified from ICD-9-CM codes*.

## Discussion

Advances in neonatal care have led to increased survival of preterm infants born at earlier GAs. However, infants born before 28 weeks GA remain at high risk of developing BPD owing to lung development being interrupted as a consequence of extremely preterm birth (6, 7). The incidence of BPD in our cohort of infants born at or before 28 weeks GA was 40.8%, which is similar to the incidences of 44.7 and 40.8% reported in other multicenter studies in the United States, where BPD was defined as oxygen use at 36 weeks PMA (2, 16). The incidence of BPD decreased dramatically with increasing birth GA: a result that is reported consistently in studies of extremely preterm infants (2, 13).

In the present study, infants with BPD had significantly longer stays in their hospital of birth compared with those without BPD and those without BPD, ROP, or IVH. Observationally, however, LOS was more closely linked (inversely) to GA at birth than to presence of BPD. Infants born at earlier GAs had longer LOS than those born at later GAs, regardless of whether or not they had BPD. Prematurity itself is a strong risk factor for long hospital stays, although a study of infants with very low birth weights reported that infants with BPD required hospital stays that were ~4 weeks longer than those for infants without BPD (9, 17).

Analysis of the total costs and charges to treat extremely preterm infants during index hospitalization showed results in line with those for the length of hospital stay. Total costs and charges were influenced more by GA at birth than by the presence or absence of BPD, with infants born before 24 weeks GA incurring substantially higher costs and charges than those born at 27–28 weeks GA. For each GA category, costs and charges for infants with BPD were higher than for those without, and were lowest for those without BPD, ROP, or IVH. Beyond index hospitalization, infants in the BPD cohort had, on average, more hospital readmissions for lung-related and other issues during their first year of life than those in the no-BPD cohort. Other researchers have reported a rehospitalization rate that is more than two times higher in preterm infants with BPD than in those without BPD (10).

Extremely preterm infants with BPD were more likely to have at least two additional hospital encounters after their index hospitalization during their first year of life compared with those without BPD, and the encounters were often related to a pulmonary issue. Among infants who returned at least twice to the hospital during their first year, pneumonia, asthma, reactive airway disease, and hypoxemia were reported more frequently in infants with BPD than in those without, whereas rates of cough and upper respiratory tract infections were similar in both cohorts. Pulmonary-related medications during the first year were used in similar percentages of patients with BPD (16.7%), without BPD (15.4%), and without BPD, ROP, or IVH (17.0%), which could indicate that medication use may be related to conditions other than BPD and might provide persistent beneficial effects. Moreover, use of these medications has not been associated with any meaningful impact on the incidence or severity of BPD (18).

We acknowledge a number of limitations in our study. The Premier Perspective database only captures data from outpatient clinics and emergency department services associated with hospitals in the database network. Rates of outpatient visits, emergency department visits, hospital readmissions, and medication use are thus likely to be underestimated in our analysis owing to patients being lost to facilities outside of the database network for follow-up. Pulmonary medication use may be further underestimated because estimations were based on reported procedural codes and charge descriptions. In addition, the presence or absence of BPD was determined from ICD-9-CM codes reported in patient files. No information was obtained on the definition used to diagnose BPD or on the severity of the disease, and results may be influenced by clinician judgement. It should also be noted that BPD and CLD are coded using the same ICD-9-CM codes. Nonetheless, the BPD rates found are consistent with those based on clinical criteria, suggesting that coding issues for BPD were not significant in this cohort.

In conclusion, extremely preterm infants with BPD had longer hospital stays and higher costs during their initial hospitalization compared with those without BPD, and had higher rates of in-hospital mortality, hospital readmission, and pulmonary morbidities during their first year of life. Strategies to improve clinical outcomes for infants with BPD are important to reduce the large health care burden associated with extremely preterm birth. Our results also indicate that increasing the GA at birth, even by 1 week, has a larger impact on health care burden than presence of BPD.

## Data Availability Statement

The data that support the findings of this study are available from Premier Perspective but restrictions apply to the availability of these data, which were used under license for the current study, and so are not publicly available. However, data are available from the authors upon reasonable request and with permission of SS.

## Author Contributions

MM, RA, AM, and SS contributed to the study design and interpretation of the results. RA, WG, and JZ contributed to data analysis and interpretation of the results. All authors provided sustained intellectual contributions to the writing of this article, revised it critically for intellectual content, and read and approved the final manuscript.

### Conflict of Interest

MM has no disclosures in relation to this study. RA, WG, and JZ are employees of Analysis Group Inc., who were paid consultants to Shire, a Takeda company, in relation to this study. AM and SS are employees of Shire, a member of the Takeda group of companies, and own stock/stock options in Takeda.

## Abbreviations

BPD: bronchopulmonary dysplasia
CLD: chronic lung disease
GA: gestational age
ICD-9-CM: International Classification of Diseases, Ninth Revision Clinical Modification
ICU: intensive care unit
IVH: intraventricular hemorrhage
LOS: length of stay
NICU: neonatal intensive care unit
PMA: postmenstrual age
ROP: retinopathy of prematurity
SD: standard deviation.
