# Supplementary material for: Health Care Burden of Bronchopulmonary Dysplasia Among Extremely Preterm Infants
Source: Front Pediatr. 2019 Dec 12;7:510. doi: 10.3389/fped.2019.00510 (PMC6921371; doi:10.3389/fped.2019.00510)
Supplement: Supplementary file 1 [file Data_Sheet_1.pdf]

Supplementary Table 1 | ICD-9-CM codes used to identify gestational age[1]

Gestational age	ICD-9-CM codes
Less than 24 completed weeks of gestation	765.21
24 completed weeks of gestation	765.22
25 to 26 completed weeks of gestation	765.23
27 to 28 completed weeks of gestation	765.24

[1]Gestational age identified based on primary or secondary ICD-9-CM diagnosis codes during the index hospitalization.

Supplementary Table 2 | ICD-9-CM codes used to identify comorbidities[1]

Comorbidities	ICD-9-CM codes
Retinopathy of prematurity (ROP)	
Stage 1	362.23
Stage 2	362.24
Stage 3	362.25
Stage 4	362.26
Stage 5	362.27
Unspecified stage	362.20
Hearing loss	389, 744.0
Strabismus	378
Blindness/low vision	369
Myopia	367.1
Amblyopia	368
Retinal detachment	361, 362.26, 362.27
Periventricular leukomalacia	779.7
General neurological dysfunction	779.0-779.2
Respiratory distress syndrome	769, 518.82, 786.09
Bronchopulmonary dysplasia (BPD)	770.7
BPD or chronic lung disease (CLD)	496, 518.89, 770.7
Apnea	770.81, 770.82, 786.03, 786.09, 780.51, 780.53, 780.57, 327.2
Pneumonia	480, 481, 482, 483, 484, 485, 486, 770.0, 487, 488
Airway obstruction, decreased lung volume	786.09
Lung hyperinflation	786.01
Lower respiratory tract infection[14]	466, 519.8
Gas trapping	786.9
Patent ductus arteriosus	747
Anemia	280, 776.5
Intraventricular hemorrhage (IVH)	772.1
Stage 1	772.11
Stage 2	772.12
Stage 3	772.13
Stage 4	772.14
Unspecified stage	772.10
Intracerebral hemorrhage	431
Necrotising enterocolitis (NEC)	777.5
Stage 1	777.51
Stage 2	777.52
Stage 3	777.53
Unspecified stage	777.50
Bacterial sepsis (early or late-onset)	038.19, 771.81, 995.91, 995.92

Bacterial meningitis	320
Hyperbilirubinemia/jaundice	277.4, 774
Hyperglycemia	790.29
Hypoglycemia	251.1, 251.2, 775.6
Congenital diaphragmatic hernia (CDH)	756.6
Pulmonary hypoplasia	748.5
Bronchitis	466.0, 490, 491
Bronchiolitis	466.1
Cough	786.2
Wheeze	786.07
Tachypnea	786.06
Dyspnea	786.0
Stridor	786.1
Reactive airway disease	519.8, 493.9
Asthma	493, V17.5,
Hypoxia	768,
Hypoxemia	770.88, 799.02
Cyanosis	782.5
RSV	079.6
Respiratory failure	518.81
Upper Respiratory Tract Infection	465.8, 465.9
Tracheostomy complications	519

[1]*Comorbidities identified based on primary or secondary ICD-9-CM diagnosis codes during the index hospitalization.*

Supplementary Table 3 | CPT and ICD-9 Procedure Codes[1]

Procedure	CPT Code	ICD-9-CM Codes
Treated by an ophthalmologist	92002, 92004, 92012, 92014	
Screening for retinopathy	92225, 92226, 92227, 92228, 92230, 92250, 99174	16.21, 95.11
Laser therapy	67105, 67145, 67228	14.34, 14.54
Cryotherapy ablation	67101, 67229	14.32, 14.52
Scleral buckling surgery	67107, 67112	14.4x
Vitrectomy	67005, 67010, 67015, 67036, 67038, 67039, 67040, 67041, 67042, 67043, 67108, 67113	14.71, 14.72, 14.73, 14.74
Angiogenesis inhibitor (bevacizumab)	J9035	
Therapeutic hypothermia	99481, 99482, 0260T, 0261T	99.81
Brain MRI	70551, 70552, 70553	88.91
Cranial CT Scan	70450, 70460, 70470	87.03
Electroencephalography (EEG)	95812, 95813, 95816, 95819, 95822, 95824, 95827, 95957, 3650F	89.14
Initial resuscitation	92950, 99465	93.93, 99.60
Supplemental oxygen therapy		V46.2, 93.96
Intubation/mechanical ventilation	31500, 94002-94004	93.90, 96.01, 96.02, 96.03, 96.7x
Surfactant therapy	94610	
Continuous positive airway pressure	94660	V46.8
Diuretics (injection of thiazide/furosemide, spironolactone, bumetanide, and acetazolamide)	J1205, J1940, S0171, J1120	
Beta-2-agonists (albuterol or levalbuterol, inhaled solutions)	J7607, J7609, J7610, J7611, J7612, J7613, J7614, J7615	
Corticosteroids (budesonide or beclomethasone, inhaled solutions)	J7626, J7627, J7633, J7634, J7622	
Prophylactic caffeine citrate	J0706	
Methylxanthine (injection of theophylline or dyphylline)	J1180, J2810, 80198	
Laparotomy	43327, 49000	54.11, 54.19
Primary peritoneal drainage	49020, 49021, 49406, 49407	
Antibiotics (ampicillin, gentamicin, metronidazole, cefotaxime, piperacillin-tazobactam, vancomycin, meropenem, amikacin, clindamycin, cefoxitin, nafcillin, cefazolin, azithromycin, and ceftazidime)	S0030, J0290, J1580, J0698, J3370, J2185, J2503, 80170, 80202, J0278, S0077, J0694, S0032, J0690, J0456, Q0144, J0713, J0714	
Inotropic and vasopressor drugs (dopamine, dobutamine, milrinone, epinephrine, and norepinephrine)	J1265, J1250, J2260, J0171	
Sildenafil	S0090	
Cranial ultrasound	76536	00.12

Inhaled nitric oxide		
ECMO	36822, 33960, 33961, 33946, 33947, 33948, 33949, 33951, 33953, 33955, 33957, 33959, 33963, 33965, 33969, 33985	39.65

[1]Procedures identified based on CPT procedure codes or ICD-9 procedure codes during the index hospitalization.
